# Unmasking Hidden Mediastinal Involvement in Papillary Thyroid Carcinoma: A Case Report on the Diagnostic Utilization of EBUS-TBNA

**DOI:** 10.1155/crpu/1770801

**Published:** 2025-07-19

**Authors:** Albatol N. Rashed, Khalid S. Alokla, Ali F. Alfayez, Luai Sallout

**Affiliations:** ^1^Pulmonary Section, Department of Medicine, King Fahad Medical City, Riyadh, Saudi Arabia; ^2^Department of Pathology and Laboratory Medicine, King Fahad Medical City, Riyadh, Saudi Arabia

**Keywords:** EBUS-FNA, mediastinal lymph node, metastasis, papillary thyroid cancer

## Abstract

The global incidence of thyroid cancer is on the rise, with papillary thyroid carcinoma (PTC) accounting for approximately 80% of all cases. The incidence of mediastinal lymph node metastases in papillary thyroid cancer ranges from 0.7% to 27%. This case report details the diagnosis of a young man with papillary thyroid cancer, where the diagnosis was confirmed using endobronchial ultrasound–transbronchial needle aspiration (EBUS-TBNA) of the mediastinal lymph nodes. EBUS-TBNA, recognized for its minimal invasiveness and high diagnostic yield, facilitated the precise evaluation and staging of mediastinal involvement. This method played a key role in identifying metastatic involvement in a case where traditional imaging and diagnostic approaches may have been insufficient. The case underscores the utility of EBUS-TBNA in managing thyroid cancer metastases, particularly in assessing rare sites of metastatic spread, and highlights the importance of this technique in the comprehensive diagnostic evaluation of thyroid cancer patients with suspected mediastinal lymph node involvement.

## 1. Introduction

Thyroid carcinoma, which constitutes less than 1% among all cancers, stands as the most prevalent form of endocrine cancer, representing about 5% of thyroid nodules [1]. Mediastinal lymph node metastases from papillary thyroid cancer (PTC) are rare but significantly impact prognosis. The common metastatic sites preceding these are neck lymph nodes and lung parenchyma [2].

Endobronchial ultrasound–transbronchial needle aspiration (EBUS-TBNA) has gained recognition as a minimally invasive yet highly effective technique, primarily employed for mediastinal staging and lung cancer diagnosis [3]. This method is less invasive compared to traditional surgical approaches and is often utilized as a first-line diagnostic tool for mediastinal lymphadenopathy of unknown origin. Additionally, EBUS-TBNA is increasingly applied beyond lung cancer diagnosis, proving effective in identifying other conditions such as sarcoidosis and tuberculosis, thereby playing a crucial role in managing a diverse spectrum of mediastinal diseases [4–6].

In addition to the widely used convex probe EBUS, the radial probe EBUS-TBNA has also been utilized in the diagnosis of metastatic primary papillary thyroid carcinoma [7].

Importantly, EBUS-TBNA is effective not only for common cancers but also for rarer malignancies, such as pulmonary large-cell neuroendocrine carcinoma [8] and ovarian carcinoma [9]. We recently published a case report as well on EBUS-TBNA diagnosing metastatic glioblastoma [10]. This underscores EBUS-TBNA's versatility in the realm of cancer diagnosis.

## 2. Case Summary

**Figure 1:**
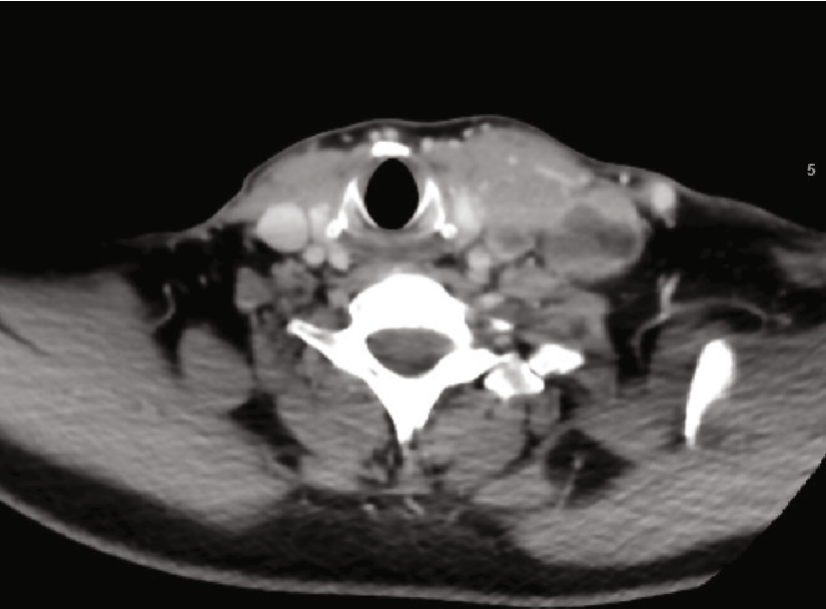
CT of the head and neck showing thyroid mass.

A 49-year-old gentleman, who had no known chronic medical illnesses, presented with a neck mass that had been enlarging over a year, showing no features suggestive of thyroid dysfunction. On clinical examination, he was found to have an enlarged thyroid gland and a palpable left cervical lymph node. Blood investigations showed that thyroid-stimulating hormone was 2.54 mIU/L (0.3500–4.9400 mIU/L), with free T4 at 13 pmol/L (9.00–19.00 pmol/L), intact parathyroid hormone at 2.30 pmol/L (1.60–7.20 pmol/L), and thyroid antibody at 418 IU/mL (< 4.00 IU/mL). A CT of the head and neck revealed a left thyroid mass measuring 8 × 6 × 4 *cm*, with multiple enlarged left cervical lymph nodes (Figure 1). He was diagnosed with PTC via fine needle aspiration. In March 2022, the patient underwent a total thyroidectomy with left lateral neck dissection and received radioactive iodine ablation in June 2022, 150 mCi with a Thyrogen injection, and was initiated on levothyroxine replacement therapy. Histopathological examination confirmed metastatic involvement of the left cervical lymph nodes at Levels III and IV. There was also evidence of extrathyroidal extension into the adjacent muscle, consistent with invasive carcinoma. Based on these findings, the final pathological stage was T2N1M1.

**Figure 2:**
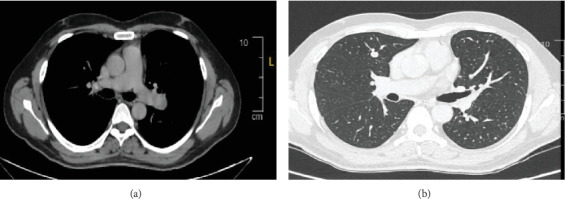
(a) Contrast-enhanced CT chest showing a left hilar lymph node measuring 2.5 × 2.2 *cm*. (b) Multiple pulmonary nodules, the largest of which is located in the right middle lobe, measuring approximately 9 mm.

**Figure 3:**
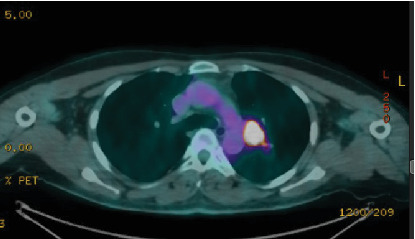
Whole-body PET scan showing FDG-avid left hilar lymphadenopathy.

**Figure 4:**
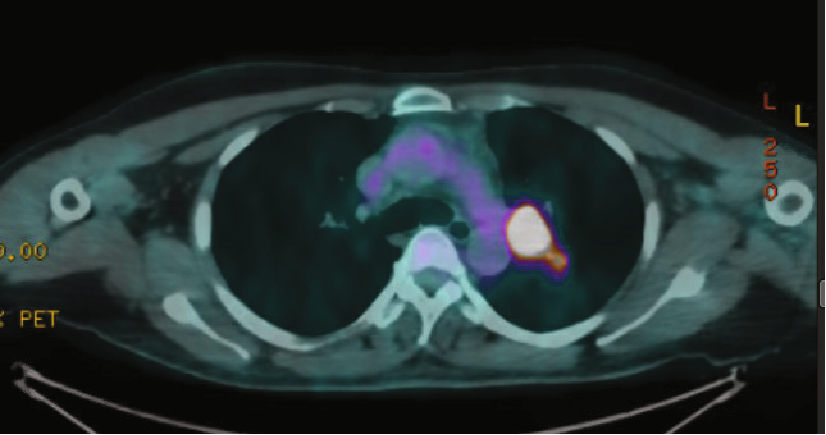
Interval morphologic progression of the previously noted hypermetabolic left hilar lymphadenopathy.

Postoperative images, including a CT chest with intravenous contrast in July 2022, showed diffuse bilateral variable-sized scattered pulmonary nodules and bilateral hilar nodal metastasis. The left hilar lymph node was noted measuring 2.5 × 2.2 *cm*, with another right hilar lymph node mildly enlarged at about 1 cm. A small prevascular space lymph node measured 6 mm in diameter (Figure 2a,b). A subsequent whole-body PET scan did not show FDG avidity within the thyroidectomy bed to suggest residual or recurrent disease, but there were multiple bilateral variable-sized pulmonary nodules with some associated mild FDG activity (SUV max 2.9). Intensely, FDG-avid left hilar lymphadenopathy was noted on PET-CT, with a maximum standardized uptake value (SUV max 17), raising concern for metastatic involvement, as an SUV greater than 2.5 is typically considered suspicious for malignancy (Figure 3). As the patient had received radioactive iodine ablation 2 months before the image, a follow-up PET scan was repeated after 6 months and showed an interval metabolic progression of the multiple bilateral variable-sized pulmonary metastatic nodules, interval morphologic progression of the previously noted hypermetabolic left hilar lymphadenopathy, and no other suspicious FDG-avid disease elsewhere in the body (Figure 4).

**Figure 5:**
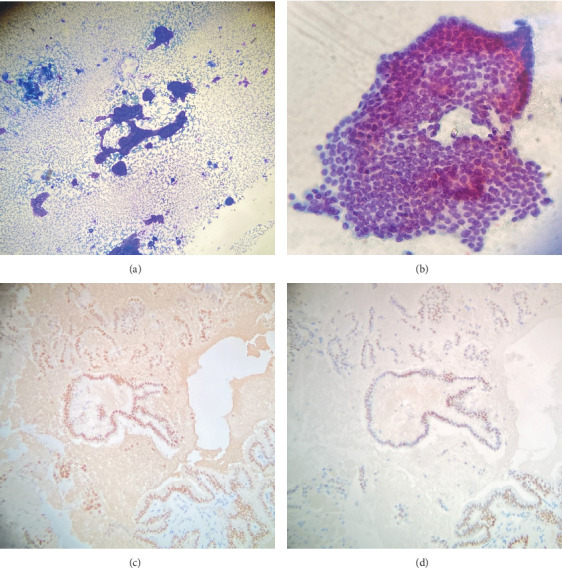
(a–d) Histopathology of the EBUS-FNA from the left hilar lymph node. (a) Direct, air-dried (Diff-Quik) smear showing branching papillary neoplastic groups at low magnification. (b) On high-power magnification, cells have enlarged overlapping nuclei with irregular contours, intranuclear inclusions, and nuclear grooves. (c, d) The neoplastic cells are strongly positive for (c) TTF-1 and (d) PAX-8; consistent with metastasis from a thyroid origin.

The patient was referred to our lung nodule clinic for assessment of the hilar lymphadenopathy and lung nodules. He had not developed any new onset respiratory symptoms or B symptoms (drenching night sweats, loss of more than 10% of body weight, or fever). An endobronchial ultrasound with fine needle aspiration was performed on the left hilar lymph node (station 10 L), which measured approximately 30 mm in size. Four transbronchial needle aspiration (TBNA) passes were done with the intention of diagnosing rather than staging, given the medical history and the radiological presentation suggesting metastasis. The rapid on-site examination (ROSE) showed follicular cells, and the histopathology examination confirmed the presence of malignant cells, which later tested positive for immunostaining for TTF-1 and PAX 8, consistent with the diagnosis of metastatic papillary thyroid carcinoma (Figures 5a, 5b, 5c, and 5d]. Given the result of the FNA and the progressive enlargement of mediastinal lymph nodes, the patient was referred to oncology for systemic therapy. A multidisciplinary tumor board meeting was held, and the consensus was to initiate a tyrosine kinase inhibitor if there was evidence of disease progression exceeding 20% of tumor size or if the patient became symptomatic. Molecular testing revealed a positive BRAF 600 mutation, and the patient was started on lenvatinib, which was gradually titrated to 20 mg daily. The treatment was well tolerated with no significant side effects reported.

## 3. Discussion

In a retrospective review, a total of 2292 patients with thyroid cancer were treated at KFSH&RC, Riyadh, Saudi Arabia, from 2000 to 2010. Thyroid cancer constitutes about 9% of all malignancies and 12% of all female malignancies at KFSH&RC, which is significantly higher compared to the United States, where thyroid cancer represents only 2.9% of all malignancies and 4.6% of all female malignancies [11]. Papillary adenocarcinoma was the most common histological subtype followed by papillary carcinoma, the follicular variant [11, 12]. About 48% of patients presented in the localized stage and 7.6% with distant metastasis [11]. In Saudi Arabia, PTC presents in an advanced stage, especially in males as manifested by the large primary tumor size, advanced pathologic staging, and distant metastases at the time of presentation [12].

While distant metastasis in PTC remains rare, understanding its dynamics is crucial for effective treatment planning. The cervicocentral compartment typically presents as the most common metastasis site, with studies documenting its prevalence. In a study of 35 patients diagnosed with PTC, mediastinal lymph node metastasis was observed in only one case [13].

Papillary thyroid carcinoma has the potential to metastasize to nearly all mediastinal lymph nodes, with the exception of stations 3P, 4L, and 7 [14]. The EBUS-TBNA technique is particularly valuable in this context due to its ability to access and sample from multiple mediastinal lymph node stations effectively. EBUS-TBNA can reliably sample from stations such as 2R, 2L, 4R, 4L, 7, 10, and 11, which are critical areas for assessing the spread of malignancies within the chest.

A more extensive retrospective study at Fudan University Shanghai Cancer Center, covering 2008 to 2015, identified mediastinal lymph node metastases in 73 of 17,745 patients who underwent surgery for thyroid cancer. Among these, the majority, 82.2%, had papillary thyroid carcinoma, while 16.4% had medullary thyroid carcinoma and 1.4% had anaplastic thyroid carcinoma [15].

Current research indicates no definitive consensus on the risk factors for distant metastasis of papillary thyroid carcinoma. However, a study involving 392 patients with mediastinal lymph node–positive PTC at a tertiary surgical center in Germany identified poor tumor differentiation and the presence of distant metastases as significant predictors of mediastinal lymph node involvement [16].

A 2016 prospective observational study further investigated risk factors for superior mediastinal lymph node metastasis, finding that despite a clinically negative preoperative evaluation, metastasis could still be anticipated in patients with PTC tumors larger than 1 cm, pretracheal lymph node metastasis, multiple lateral metastases, and those undergoing revisional surgery [17]. In contrast, previously considered predictive factors like tumor size, lymphovascular invasion, and perithyroidal extension were not significantly associated with an increased risk of metastasis [17].

Despite numerous investigations into the efficacy of EBUS-TBNA in identifying intrathoracic metastases from extrathoracic cancers, there remains limited data on its effectiveness in detecting mediastinal or hilar lymph node metastases originating from papillary thyroid carcinoma [7, 18–20]. The results and safety of EBUS-TBNA in these case reports underscore its potential as a pivotal, minimally invasive diagnostic tool for evaluating mediastinal lymph node enlargement in patients with papillary thyroid carcinoma.

EBUS-TBNA is a minimally invasive and highly effective method for sampling mediastinal and hilar lymph nodes in lung cancer, with sensitivity between 81% and 95% and 100% specificity. In comparison, mediastinoscopy—though considered the gold standard—has slightly lower sensitivity (75%–90%), the same specificity (100%), but is more invasive and limited to certain lymph node areas [21]. CT-guided percutaneous biopsy is effective for accessible lung or mediastinal masses, with 70%–90% sensitivity and 95%–100% specificity. Though all three techniques are accurate, EBUS-TBNA is preferred for the initial evaluation due to its real-time guidance and minimally invasive nature [21, 22].

In a 5-year study by Yasufuku et al., EBUS-TBNA was found to be highly effective for diagnosing mediastinal masses of unknown cause (not related to lung cancer), with a 93.6% overall diagnostic rate. It had a yield of 87.5% for malignant and 96.0% for benign conditions, and importantly, no complications were reported [23].

Given the rarity and significant implications of mediastinal lymph node metastasis in thyroid cancer, it is advisable to prioritize the investigation of any mediastinal lymph node enlargements in such patients using EBUS-TBNA as a primary diagnostic approach. This strategy enhances the management and treatment planning for affected individuals, providing a crucial intervention point that may significantly influence patient outcomes.

In conclusion, while the management of mediastinal lymph node metastasis in thyroid cancer remains challenging, leveraging advanced diagnostic tools like EBUS-TBNA in conjunction with a thorough understanding of associated risk factors can significantly enhance patient care and prognosis. To the best of our knowledge, there are no previously published cases from Saudi Arabia that describe the use of EBUS-TBNA for the staging of papillary thyroid carcinoma.

## Data Availability

The data that support the findings of this study are available from the corresponding author upon reasonable request.
